# The Role of Bone in Muscle Wasting

**DOI:** 10.3390/ijms22010392

**Published:** 2020-12-31

**Authors:** Gordon L. Klein

**Affiliations:** Department of Orthopaedic Surgery and Rehabilitation, University of Texas Medical Branch, Galveston, TX 77555-0165, USA; gordonklein@ymail.com

**Keywords:** bone resorption, inflammation, calcium, transforming growth factor β, muscle atrophy

## Abstract

This review describes the role of bone resorption in muscle atrophy as well as in muscle protein anabolism. Both catabolic and anabolic pathways involve components of the proinflammatory cytokine families and release of factors stored in bone during resorption. The juxtaposition of the catabolic and anabolic resorption-dependent pathways raises new questions about control of release of factors from bone, quantity of release in a variety of conditions, and relation of factors released from bone. The catabolic responses involve release of calcium from bone into the circulation resulting in increased inflammatory response in intensity and/or duration. The release of transforming growth factor beta (TGF-β) from bone suppresses phosphorylation of the AKT/mTOR pathway and stimulates ubiquitin-mediated breakdown of muscle protein. In contrast, muscle IL-6 production is stimulated by undercarboxylated osteocalcin, which signals osteoblasts to produce more RANK ligand, stimulating resorptive release of undercarboxylated osteocalcin, which in turn stimulates muscle fiber nutrient uptake and an increase in muscle mass.

## 1. Introduction: Bone Resorption

Bone resorption, the process by which bone is broken down to liberate products needed by the body’s metabolism, most prominently, but not exclusively, calcium, is incompletely understood. However, what we do know involves most prominently three bone cell types. The osteoblast is the cell which produces new matrix in the form of type I collagen, which undergoes a process of crystal formation ending up as hydroxyapatite matrix. It also serves as a receptor for parathyroid hormone (PTH) and is affected by proinflammatory cytokines, such as interleukin (IL)-1β and IL-6, which stimulate the osteoblast to synthesize the ligand of the receptor activator of nuclear transcription factor ϏB (NFϏB), also known as RANK ligand or RANKL. This product stimulates marrow precursor cell differentiation into osteoclasts resulting in increased bone resorption and serves as one known coupling factor between bone formation and resorption. The *osteocyte*, the cell type which becomes the ultimate fate of osteoblasts, serves as the bone’s mechanosensor, connected to other osteocytes by means of a reticular network. The osteocyte network senses mechanical stress and strain and can sense the weak areas of bone, the areas in which there may be microcracks, and is the largest producer of RANKL in the body. Thus, the osteocyte is capable of stimulating resorption of the weak areas in the bone to be replaced by newly synthesized calcified matrix. The *osteoclast* is the result of differentiation of the bone marrow granulocytic precursor cells ending up as a producer of enzymatic products, most prominently caspase, that can erode the weakened bone and produce substances called clastokines, most notably sphingosine kinase 1, that can stimulate osteoblast migration to the eroded area and, aided by biphasic calcium phosphates such as hydroxyapatite and β-tricalcium phosphate, aid in new bone formation [1]. Furthermore, Ikebuchi et al. [2] have recently demonstrated that the receptor activator of NF₭B (RANK) produced by osteoclasts binds with osteoblastic RANKL to activate osteoblastic Runx2 transcription factor, thus promoting osteoblastogenesis and contributing to the normal linkage of bone resorption to formation. Any other factors that influence the coupling of osteoblasts and osteoclasts remain to be identified.

### 1.1. Clinical Conditions That Can Cause Resorptive Bone Loss

A variety of clinical conditions can cause resorptive bone loss. The most common known condition is hyperparathyroidism, as all students learn in medical school that low circulating ionized calcium results in parathyroid calcium-sensing receptor-mediated stimulation of PTH secretion with consequent bone resorption and liberation of bone calcium to normalize circulating calcium.

Another condition that results in resorptive bone loss is Paget’s disease. In this condition, the precise cause of the resorptive bone loss is unclear though thought to be genetically linked. It tends to run in families of Northern European ancestry, although a viral etiology has also been suggested.

Chronic kidney disease may also cause resorptive bone loss, since the more severe forms are associated with secondary hyperparathyroidism triggered by the kidney’s inability to excrete phosphate.

Acute burn injury and cancer metastases to bone, most commonly breast and lung, result in inflammatory reactions which stimulate resorptive bone loss [3,4]. In addition, burn injury creates a stress response causing the increased endogenous synthesis of glucocorticoids by the adrenal glands leading to hyperadrenocorticism and urinary cortisol excretion 3–8-fold above the upper limits of normal [5]. The liberation of calcium and other products of bone serve particular metabolic needs following the acute disruption of normal body metabolism [6].

### 1.2. Factors Liberated by Bone Resorption: What We Know and Do Not Know

Calcium is the most widely documented factor liberated by bone resorption. While previously thought to exclusively maintain normal circulating calcium concentration, more recent data suggests that it has an effect on systemic inflammation. The earliest report is from Rossol et al. [7] in 2012, who reported that extracellular calcium can upregulate the nod-like receptor (NLR) P3 inflammasome, which acts on the monocytes and macrophages of the innate immune system, stimulating them to produce more IL-1. This effect is modulated by the calcium-sensing receptor (CaSR). Subsequently, in 2016, Klein et al. [8] reported that cultures of peripheral blood mononuclear cells from normal adult volunteers produced chemokines in quantities that varied either directly or inversely with the amount of calcium in the culture medium. Taken together, these reports provide evidence supporting an effect of calcium liberated from bone on prolongation or intensification of the inflammatory process, or perhaps on both.

Clinical experience with burn patients is consistent with the inflammatory response being affected by extracellular calcium. Thus, pediatric burn patients can upregulate the parathyroid CaSR in response to both IL-1 and IL-6 [9,10,11] resulting in hypocalcemic hypoparathyroidism [12]. In contrast, adult burn patients maintain normal to slightly elevated circulating calcium and PTH concentrations [13,14], indicating that they have lost the ability to upregulate the CaSR in response to proinflammatory cytokines. Adult patients experiencing thermal injury of the same extent and severity as pediatric patients are reported to suffer a comparatively greater burn morbidity [15], suggesting that the severity of their inflammatory response is greater or more prolonged than their pediatric counterparts.

Phosphate and magnesium are also released by resorbing bone. We know this also from experience with pediatric burn patients when muscle protein kinetics were studied using stable isotopes. Typically, pediatric burn patients who receive an intra-arterial infusion of unlabeled amino acids use those amino acids to increase muscle protein synthesis in an attempt to compensate for the increase in muscle protein breakdown that occurs during the systemic inflammatory and stress-induced hypermetabolic response. When an antiresorptive agent such as a nitrogen-containing bisphosphonate was given to these patients, muscle protein synthesis actually fell [16], suggesting that bone was the source of phosphate and magnesium needed for the ATP synthesis required by muscle to support new protein synthesis [6].

Another important factor produced by resorbing bone is transforming growth factor (TGF)-β. As noted in a recent review by Morikawa [17], TGF-β1-3 are multifunctional polypeptides present in most tissues. They engage in signal transduction through transmembrane serine/threonine kinase receptors, and then through phosphorylation of Smad 2/3, the complexes translocate to the cell nucleus, where they regulate the expression of various target genes. TGF-β is produced by osteoblasts and is stored in the extracellular matrix of bone [17]. Dallas et al. [18] reported that the latent TGF-β stored in the extracellular matrix is released by osteoclastic proteolysis of the latent TGF-β binding protein 1 and this process may be mediated by matrix metalloproteinases (MMP)-2 and MMP-9.

An additional factor known to be released from bone during resorption is undercarboxylated osteocalcin. Osteocalcin is a protein synthesized by osteoblasts, gamma carboxylated by vitamin K, and found in bone matrix. Originally, it was considered as a marker of osteoblast differentiation until the work in the laboratories of Karsenty and others demonstrated that its metabolically active form, the undercarboxylated osteocalcin, is a potent regulator of glucose metabolism through its Gprc6A receptor on pancreatic islet cells with a specific effect on muscle glucose uptake, as we shall see [19].

All factors so far listed are released from bone on resorption and all have specific effects on muscle protein catabolism or anabolism. These will be discussed in further detail below. Other factors released and their metabolic consequences remain to be described. Thus, the picture of bone factor release with effects on muscle is to date by no means complete. The known factors involved in resorptive release from bone and which play a role in the size of muscle mass are summarized in Table 1.

### 1.3. Bone Factor Release and Muscle Catabolism

Of the above-listed resorption-released bone factors, at least two—calcium and TGF-β—have catabolic effects on muscle. The calcium effect is indirect. If the patient suffers from an underlying inflammatory condition, the release of calcium from resorbing bone has the capacity to intensify and/or prolong the inflammatory response, augmenting or perpetuating the production of proinflammatory cytokines. While the complete mechanism for cytokine effect on muscle protein breakdown is still under investigation, the catabolic effect of proinflammatory cytokines on muscle has been well documented [20].

The first report that TGF-β released by bone resorption was catabolic to muscle came from Waning et al. [3] in the laboratory of Theresa Guise in Indiana. In mouse and human studies of breast cancer metastatic to bone, they learned that bony metastases caused inflammation-related resorption with release of TGF-β and consequent upregulation of NADPH oxidase 4 (nox4) resulting in elevated oxidation of muscle proteins, including the ryanodine receptor calcium release channel 1 (RyR1). The resulting calcium leak caused a lower intracellular calcium signaling than required for normal muscle signaling. Inhibiting RyR1 leakage, TGF-β signaling or release from bone, or nox4 activity improved muscle function in mice with bone metastases. Humans with breast- or lung-cancer-related bone metastases also exhibited oxidized skeletal muscle RyR1 receptors not seen in normal muscle. Furthermore, a mouse model of Camurati–Engelmann disease, a metabolic bone disorder with increased TGF-β activity, demonstrated similar findings.

At the same time, Borsheim et al. [16] reported muscle protein kinetics results in pediatric burn patients who participated in a randomized, controlled, double-blind study of the antiresorptive nitrogen-containing bisphosphonate pamidronate, evaluating its effect on resorptive bone loss in burns. They found that subjects who received pamidronate compared with saline placebo had not only preserved bone mass but also reduced muscle protein synthesis and breakdown, leaving them with a positive muscle protein balance compared with the negative balance normally observed under these circumstances. These differences were also found when examining muscle fiber diameter from biopsies taken at the same time as the kinetic studies were performed and also in peak torque of lower extremity muscles at 9 months postburn. While there was no immediate explanation available for this finding, Pin et al. [21] investigated serum from patients who participated in the abovementioned clinical trial of bisphosphonates, specifically examining their effect on myotube development in cultures of murine C2C12 myoblasts. In this study, myoblast cultures containing serum from burn subjects who received placebo demonstrated significantly reduced myotube size compared with normal, while cultures containing serum from subjects treated with pamidronate demonstrated partial rescue of myotube size.

Furthermore, myoblast cultures containing serum from burned subjects receiving placebo showed significantly increased concentrations of ubiquitin, a key protein in the ubiquitin ligase catabolic pathway, and reduced phosphorylation of the anabolic Akt/mTor pathway, whereas cultures with serum from subjects receiving pamidronate demonstrated significantly reduced concentrations of ubiquitin compared with placebo and significantly increased phosphorylation of the Akt/mTor anabolic pathway. When the myoblast culture experiments were repeated with the addition of anti-TGF-β1-3, myotube size of the placebo controls was rescued to the magnitude of those treated with bisphosphonates, while the myotube size of the bisphosphonate-treated patients did not change significantly in culture with the addition of the antibody. While the study of Pin et al. did not examine muscle protein oxidation or the state of the ryanodine receptor, both the work of Waning et al. [3] and of Pin et al. [21] support the liberation of TGF-β from bone on resorption and its deleterious effects on muscle protein.

Moreover, adults with bony metastases from breast or lung cancer, and previously normal healthy children exposed to burn injury are two distinct groups of patients exhibiting a similar mechanism of muscle loss in the face of two distinct conditions with vastly differing etiologies, giving rise to the possibility that this mechanism is basic to the control of muscle mass by bone.

Essex et al. [22] reported that treatment with zoledronate, an antiresorptive bisphosphonate, is able to prevent muscle wasting in normal healthy mice given the chemotherapeutic agent cisplatin, while Hain et al. [23] demonstrated similar effects of zoledronate treatment with use of carboplatin in mice with breast cancer metastatic to bone. Both mouse studies showed that chemotherapeutic agents themselves can cause inflammation-induced bone resorption and muscle wasting and it was the bisphosphonate treatment in each case that prevented the bone resorption and preserved the muscle mass.

Still another set of studies by Bonnet and colleagues [24] in Geneva, utilizing the monoclonal antibody to RANKL, denosumab, found that its administration over three years to postmenopausal osteoporotic women resulted in improved appendicular lean mass and grip strength, while the use of bisphosphonates did not. Further, denosumab administration to mice improved muscle strength, insulin sensitivity, and glucose uptake. Based on the differences in findings from bisphosphonates, the effects of denosumab on muscle strength were attributed to improved glucose utilization. While this is clearly possible, denosumab, like the bisphosphonates, is an antiresorptive agent, and the antiresorptive effects on the release of TGF-β from bone have not been evaluated under this specific set of experimental conditions. Taken together, this entire group of studies has already raised the possibility that antiresorptive agents can be used therapeutically to prevent bone-resorption-induced muscle wasting in patients with clinical conditions involving high rates of bone resorption. To date, there have been no clinical trials in this area.

A third factor released during bone resorption, undercarboxylated osteocalcin, has an anabolic effect on muscle. These data come mainly from the work of Gerard Karsenty’s laboratory at Columbia. Because osteocalcin concentration in serum falls with age in humans, monkeys, and mice, Mera et al. [25] studied osteocalcin knockout mice and 9-month-old wild-type mice receiving exogenous osteocalcin for 28 days. They showed that osteocalcin signaling was necessary to maintain muscle mass in older mice because osteocalcin stimulates muscle protein synthesis without affecting breakdown rate. Furthermore, the osteocalcin supplement in the aging wild-type mice was enough to increase muscle mass. In addition, osteocalcin increases nutrient intake and catabolism in myofibers and increases IL-6 expression in muscles during exercise [19]. IL-6 has pleiotropic effects in muscle, among which it increases the generation of osteocalcin [19]. A recent publication by Chowdhury [26] showed that mice lacking the osteocalcin receptor only in osteoblasts have reduced exercise comparable to mice lacking muscle generation of IL-6 and that muscle-derived IL-6 produces increased nutrient uptake by myofibers in an osteocalcin-dependent manner. They concluded that muscle-derived IL-6 stimulates osteoblasts to make more RANKL, which will stimulate increased bone resorption with consequent liberation of more undercarboxylated osteocalcin, which will then increase muscle exercise capacity.

### 1.4. Muscle Factors Influencing Atrophy

While this review focuses on factors released by bone resorption that affect muscle wasting, it is important to mention two muscle-derived factors that may influence muscle mass possibly through interaction with bone. These are myostatin and irisin.

Myostatin is a member of the TGF-β superfamily but a member of a different subfamily than the TGF-β molecule itself, namely, the activin/myostatin subfamily [27]. It is encoded by the MSTN gene, is synthesized in myocytes, and serves to limit glucose uptake and adipogenesis of myofibers. [27]. Activation of TGF-β differs from myostatin activation in that myostatin lacks a latent binding protein that aids in the liberation of TGF-β from bone matrix as previously mentioned [18]. Myostatin interacts with extracellular matrix proteins LTBP3 and perlecan in contrast to LTBP1, although both TGF-β and myostatin signal by phosphorylation of Smad 2/3 [27]. Myostatin can also signal via noncanonical pathways such as ERK, JNK, and p38 MAP kinase. As we have seen with TGF-β and osteocalcin, myostatin, though produced in muscle, stimulates osteoblastic RANKL to augment osteoclastogenesis in bone through SMAD-2-dependent nuclear factor of activated T cells, or NFATc1 [28]. At least, this is the case for myostatin expression in the joints of patients with rheumatoid arthritis [28]. The increased resorption could also add a complicating factor of TGF-β release to influence the size of the muscle mass. Also of interest is the role of myostatin in muscle glucose metabolism. Thus, a study of patients by Assyov et al. [29] found that in those with abnormalities of glucose handling, serum concentrations of myostatin were highest in those with type 2 diabetes, less elevated in those with prediabetes, and lowest in those with normal glucose control. While there have been attempts to inhibit myostatin as a means of preventing muscle atrophy, Mouisel et al. [30] reported that that in a mouse study, lack of myosin profoundly impairs oxidative phosphorylation-dependent muscle function, converting muscle metabolism from aerobic to anerobic with reduced mitochondrial respiration and lactic acidosis associated with exercise. Therefore, while myostatin inhibition may increase muscle size, the muscles are much less likely to sustain exercise, undergoing fatigue rapidly. Thus, this finding would render impractical myostatin inhibition to prevent muscle atrophy.

Another hormone primarily expressed in muscle is irisin, a fibronectin-III-containing protein that promotes glucose uptake by muscle fibers as well as the browning of white adipose tissue [31]. In addition, irisin also affects osteocytes via the αVβ5 integrin, binding to which can reduce ovariectomy-induced bone resorption as well as osteocyte production of sclerostin, effectively reducing the rate of bone turnover [32]. Irisin actions, however, do not appear to be mediated by proinflammatory cytokines. While the measurement of serum concentrations of irisin are variable, depending on the assay used, there is general agreement that acute but not chronic exercise of skeletal muscle releases increased amounts of irisin into the circulation [32]. Also of note is that the TGF-β effector protein, SMAD3, is capable of suppressing irisin in muscle. Thus, irisin appears to have some anabolic activity in muscle, possibly reducing bone resorption. While unknown, it would be of interest to speculate on the relationship between undercarboxylated osteocalcin and irisin inasmuch as both have anabolic activities in muscle.

## 2. Discussion and Summary

A list of bone- and muscle-secreted factors that affect muscle and bone is given in Table 1.

**Figure 1:**
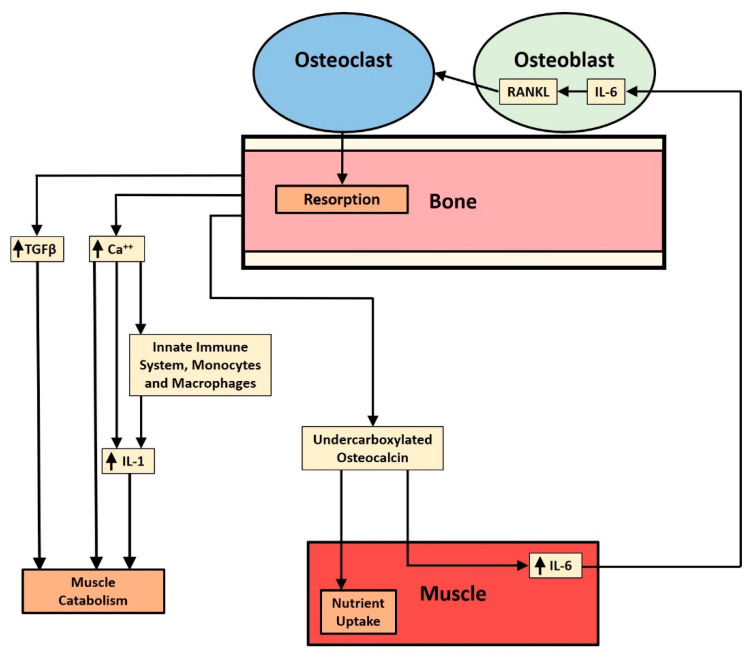
Schematic diagram illustrates the three pathways affecting muscle that are stimulated by bone resorption and mediated by proinflammatory cytokines. From the outside in, there is TGF-β release, ionized calcium release, and undercarboxylated osteocalcin release. The TGF-β and ionized calcium are catabolic, while the osteocalcin pathway has an anabolic component. The purpose of the juxtaposition of the three pathways is to raise the questions of how release is controlled and how the quantities of each substance released are regulated. TGF-β suppresses the anabolic AKT/mTOR pathway in muscle while upregulating the catabolic ubiquitin ligase pathway. Ionized calcium stimulates the NLRP3 inflammasome to produce IL-1 and mononuclear cells to release more chemokines in order to prolong or intensify inflammation and muscle loss. Osteocalcin stimulates muscle to take up more nutrients and to increase production of IL-6, which stimulates osteoblasts to make RANK ligand, stimulating osteoclastogenesis and release of undercarboxylated osteocalcin from bone.

Components of the inflammatory response appear to be active in the three known catabolic and anabolic effects that bone resorption products have on muscle wasting or muscle mass accretion. As shown in Figure 1, bone resorption and release of specific factors figure in each of the three mechanisms described: the release of calcium and TGF-β from bone matrix involved in the muscle wasting effect and the release of undercarboxylated osteocalcin involved in the anabolic effect. The components of muscle that may affect muscle mass, namely, myostatin and irisin, do not appear to be affected by the proinflammatory cytokine components of systemic inflammation, although NFATc1 may be important in the functioning of myostatin.

These observations raise questions about the nature of factor release from bone. Are specific factors, such as undercarboxylated osteocalcin or TGF-β, specifically targeted for release depending on the original trigger for the bone resorption? Or, does bone resorption indiscriminately release all products of resorption in the same ratio and at the same rate with utilization dependent on the underlying metabolic demands? To cite an example, in pediatric burn injury, circulating osteocalcin is reduced [33] as bone mass is reduced [4,34]. Thus, the release of calcium and TGF-β may be greater than that of undercarboxylated osteocalcin. Moreover, as indicated at the beginning of this discussion, there are likely many more examples of bone–muscle crosstalk that influence muscle wasting or accrual. Moreover, interactions of the musculoskeletal system with adipose tissue and the autonomic as well as the central nervous system must be better understood before a complete picture emerges of all the factors involved in muscle wasting.

Nevertheless, knowing what we do about factors released during bone resorption and their effects on muscle mass, it is possible to consider clinical trials to begin to address the problem of muscle wasting together with bone loss, or osteosarcopenia. An example of a clinical trial would be the use of antiresorptive agents to block resorptive release of bone TGF-β, which has been shown to work experimentally in both metastatic bone disease from breast cancer and in pediatric burn injury. A large-scale trial of this potential therapeutic option has not yet been undertaken in pediatric burn injury, partly due to unfamiliarity with the pharmacology of this class of drugs by clinicians caring for burns patients. As we learn more about the release of factors affecting muscle mass from bone and elsewhere in the body, additional therapeutic options may become available.

## Figures and Tables

**Table 1:** Bone and muscle factors that affect muscle mass.

Factor	Source	Function
Calcium	bone	prolongs or intensifies inflammation, leading to increased bone and muscle wasting
TGF-β	bone	promotes muscle protein catabolism and suppresses anabolism
Osteocalcin	bone	promotes muscle fiber glucose and nutrient uptake, anabolic
Myostatin	muscle	inhibits muscle fiber uptake of nutrients, inhibits fiber growth
Irisin	muscle	stimulates muscle fiber nutrient uptake, decreases bone turnover, anabolic

## Data Availability

Not applicable.
